# Rationalized design to explore the full potential of PLGA microspheres as drug delivery systems

**DOI:** 10.1080/10717544.2023.2219864

**Published:** 2023-06-05

**Authors:** Rebeca Martinez-Borrajo, Patricia Diaz-Rodriguez, Mariana Landin

**Affiliations:** a Departamento de Farmacología, Farmacia y Tecnología Farmacéutica, Grupo I + D Farma (GI-1645), Facultad de Farmacia, Universidade de Santiago de Compostela, Santiago de Compostela, Spain; b Instituto de Investigación Sanitaria de Santiago de Compostela (IDIS), IDIS Research Institute, Santiago de Compostela, Spain; c Instituto de Materiais da Universidade de Santiago de Compostela (iMATUS), Santiago de Compostela, Spain

**Keywords:** Machine learning, neurofuzzy logic, artificial intelligence, microparticles, emulsion-solvent evaporation, PLGA

## Abstract

Polymeric microparticles are widely used as drug delivery platforms either alone or embedded in more complex structures for regenerative medicine. Emulsion-solvent evaporation is the most extensively used technique for microparticles preparation. Despite the apparent simplicity of this method, there is no general procedure for producing microparticles of predictable characteristics (particle size, size distribution, encapsulation efficiency, and drug loading). Hybrid systems such as neurofuzzy logic allow identifying relationships between inputs and outputs, expressing the generated mathematical models through rules in linguistic format. In this work, the relationships between the variables involved in the emulsion-solvent evaporation process and the quality parameters of PLGA microparticles as drug delivery systems were established. Neurofuzzy logic software was able to generate models of high predictability (> 85%) for the microspheres properties namely particle size, size distribution, encapsulation efficiency and drug loading. Moreover, the generated sets of IF-THEN rules allowed to dictate general guidelines to better select the PLGA microparticles formulation parameters. This approach would be of great interest as a starting point to set-up protocols for the development of PLGA microparticles obtained by emulsion-solvent evaporation for many applications.

## 1. Introduction

Microparticles have been extensively explored in regenerative medicine either alone or embedded in 3D constructs. Among the different microparticle types; ceramic, metallic, composite and polymeric, the last ones are of particular interest due to their degradation profiles and biocompatibility (Ogay et al., 2020). Furthermore, polymeric microparticles are suitable carriers for therapeutic molecules allowing their controlled release and the creation of intricate drug-delivery platforms, such as dual release profile systems (Oliveira & Mano, 2011). These systems provide stability to encapsulated drugs and reduce the risk of side effects associated with fluctuations in the drug concentration (Varde & Pack, 2004; Tran et al., 2011; Han et al., 2016; Mensah et al., 2019). Therefore, fulfilling the requirements for drug delivery during tissue development and repair.

Despite the profuse literature on polymeric microparticles development, there are no general protocols for producing microparticles with predictable encapsulation efficiency, particle size, size distribution, and drug loading (Park et al., 2019). Nowadays, most researchers still follow the limited trial and error strategy using different methods as emulsion-solvent evaporation, supercritical CO_2_ technology, electrospray, spray drying, microfluidics or hydrogel template (Han et al., 2016; Park et al., 2019; Henshaw et al., 2021). The emulsion-solvent evaporation method, whether single or double emulsion, is one of the most widely used strategies for small-scale microparticle development, as it is simple, inexpensive, reproducible, and compatible with a wide variety of polymers and therapeutic molecules without requiring specialized equipment (Varde & Pack, 2004; Han et al., 2016; Mensah et al., 2019; Varela-Fernández et al., 2022). The single emulsion strategy is used when the drug is medium-highly soluble in the organic phase, usually the phase containing the polymer. On the other hand, when the drug is not soluble in this organic solution a double emulsion strategy is generally required. Despite the apparent simplicity of this method, the development of new microparticulate-based systems through emulsion-solvent evaporation is challenging as the production procedure involves several operations, most of them barely described in the literature. Literature reports focused on the effects of several variables on polymeric microparticles often arrive at formulations with the desired characteristics. However, they often fail to establish general rules about how the process variables condition the characteristics of the microparticles, generating procedures with unpredictable results (Szlęk et al., 2016; Mensah et al., 2019).

PLGA (poly D, L-lactic-co-glycolic acid) is a biocompatible and biodegradable polymer approved by the Food and Drug Administration (FDA) and by the European Medicines Agency (EMA) for several medical applications. Nowadays, there are different PLGA systems on the market that are used, for example, as depots or implants (Operti et al., 2021). PLGA allows the encapsulation of hydrophilic and hydrophobic drugs, and biomacromolecules such as proteins, nucleic acids, or growth factors. This polymer is commonly employed to obtain prolonged-release microparticles. As an example, the incorporation of drug-loaded PLGA microparticles in porous scaffolds has been tested to promote bone and cartilage regeneration (Tran et al., 2011; Ortega-Oller et al., 2015; Gentile et al., 2016; Han et al., 2016; Szlęk et al., 2016). However, as stated before, there are no studies reflecting how the variables involved in the emulsion-solvent evaporation processes, operation conditions and composition, affect microparticles properties as particle size, size distribution, encapsulation efficiency (EE) or drug loading (DL). Identifying and understanding the critical variables in microparticle development is not an easy task. The effect of each variable is often analyzed individually, which is costly and time consuming, as well as being an inefficient procedure as it does not consider the interaction between variables. Design of experiments (DoE) such as Taguchi design, fractional factorial design or Plackett-Burman design could be good tools to solve this inconvenience, but when the number of variables is important, the difficulty in modeling the results and drawing correct conclusions becomes extraordinary (Wang et al., 2006; Mensah et al., 2019).

Artificial intelligence (AI) tools have been proposed as useful techniques for establishing general patterns by extracting knowledge from large databases. Machine learning techniques can be used to improve dosage systems formulation, establishing relationships between inputs (ingredients and operation conditions) and outputs (formulation properties), and even to optimize procedures without carrying out a single additional experiment (Rowe & Colbourn, 2003; Colbourn et al., 2011; Echezarreta-López & Landin, 2013).

Artificial Neural Networks (ANNs) are part of the AI tools. ANNs mimic the working process of the human brain, being able to deal with large databases and allowing to establish general patterns and non-linear cause-effect relationships between inputs and outputs (Wang et al., 2006; Landín et al., 2009; Colbourn et al., 2011). Recently, ANNs have been successfully used for tailoring different dosage forms as nanoparticles, topical patches, tablets or hydrogels (Rouco et al., 2018; Lefnaoui et al., 2020; Simões et al., 2020; Garcia-del Rio et al., 2021).

ANNs can be combined with other AI tools to improve their functionality. That is the case of Neurofuzzy logic (NLF), a hybrid technique that combines ANNs and fuzzy logic (Rowe & Colbourn, 2003). NLF merges the learning ability of ANNs and the linguistic rules of fuzzy logic. As a result, complex mathematical models are expressed as sets of simple IF-THEN rules, easy to understand and interpret (Rowe & Colbourn, 2003; Colbourn & Rowe, 2009).

The objective of this work is to apply machine learning techniques to generate guidelines to assist in the development of drug-loaded PLGA microparticles produced by the single emulsion-solvent evaporation technique following the quality by design (QbD) concept. This concept is essential to ensure the quality of the microparticles by controlling and understanding the effect of variables involved in the manufacturing process and their interactions (Operti et al., 2021). The influence of several composition and operation conditions on the production process and on the microparticles ‘Critical Quality Attributes’ (microparticle size and size distribution (uniformity), encapsulation efficiency, and drug loading) will be established based on experimental results using an extremely reduced experimental design for the number of variables selected. General rules will be proposed for the customization of manufacturing protocols, from which decision trees will be developed following the obtained rules for the different critical quality attributes. A manufacturing protocol will be established to obtain microspheres with desired characteristics and experimentally tested. The characteristics of the microparticles (particle size, size distribution, encapsulation efficiency and drug loading) obtained experimentally will be compared with the models estimated values to validate the procedure.

## 2. Materials and methods

### 2.1. Materials

Poly (D,L-lactide-co-glycolide) (PLGA; Resomer® RG 502H) was supplied by Evonik, Ltd. (Germany). Polyvinyl alcohol (PVA) (87%–90% hydrolyzation, Av. Mol. Wt. 30,000 Da–70,000 Da) was obtained from Sigma-Aldrich (USA). Dichloromethane of high-performance liquid chromatography (HPLC) grade was purchased from Merck (Germany). All experiments were carried out using Milli-Q^®^ water.

### 2.2. Experimental design

**Figure 1.**
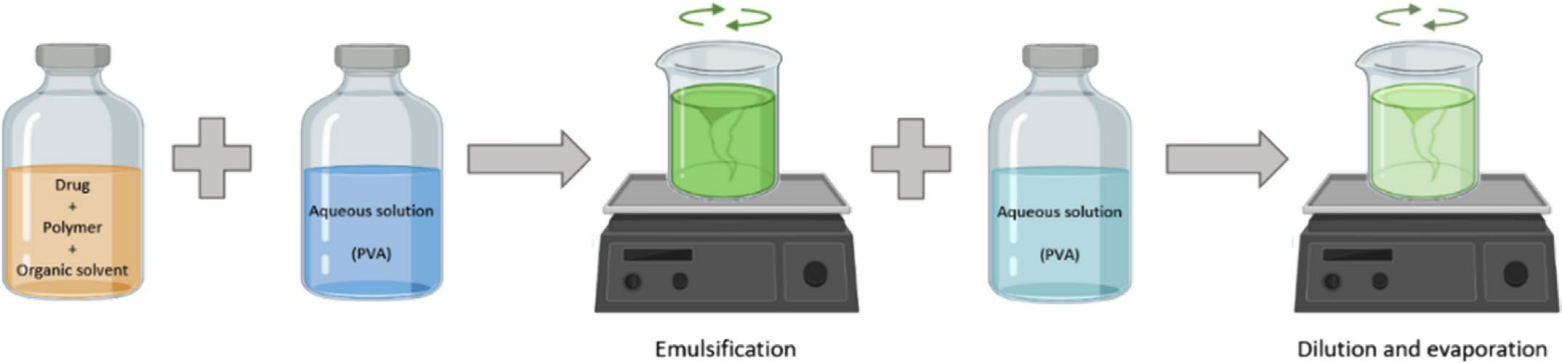
General procedure for the formation of polymeric microparticles by single emulsion-solvent evaporation process.

Polymeric microparticles were developed using the single emulsion-solvent evaporation method outlined in Figure 1. Briefly, an organic phase containing the drug (0%–2%), Resomer^®^ RG 502H (10%–20%), and 1 mL of dichloromethane (DCM) was prepared and added to an aqueous phase (0.5%–2% of PVA) using volumes of the aqueous phase between 2.5 mL and 10 mL to obtain the different O/W ratios (0.1–0.4). Both phases were mixed using a vortex (Fisherbrand, USA) at the preset speed (1,000 rpm–3,000 rpm) for a pre-established time (60 s–120 s) to form the emulsion. Subsequently, the emulsion was added to an aqueous solution (0.2% of PVA; volume between 3.5 mL to 99.0 mL), to obtain different dilution ratios (2–10) and maintained at 400 rpm using a magnetic stirrer (Fisherbrand, USA) for 2 h to reach complete dichloromethane evaporation. Finally, microparticles were filtered using a membrane disk filter (GHP, 0.45 μm, 47 mm diameter, Waters), washed repeatedly with Milli-Q^®^ water, and lyophilized.

A reduced experimental design, shown in Table 1, was stablished by DataForm^®^v.3.1 software (Intelligensys Ltd., UK) using a balanced density method for seven variables relative to the composition and operation conditions during the microparticles production process at 3 levels with a minimum pattern of 8: drug concentration ([Drug]), polymer concentration ([PLGA]), surfactant concentration during the emulsification process ([PVA]), organic/aqueous phase ratio (O/W ratio), stirring speed during the emulsification process (Speed), the stirring time (Time), and the total volume/emulsion volume ratio (Dilution ratio) during solvent evaporation. A hydrophobic model drug (LogP = 5.2, MW ≈ 300 Da) was used for the whole experimental design as well as for the further model validation step. The variables and their limits listed in Table 1 were selected following data in the literature (Mensah et al., 2019).

**Table 1.** Experimental design established by DataForm^®^ using a balanced density method for 7 variables and minimum patterns of 8.

Sample	[Drug] (%)	[PLGA] (%)	[PVA] (%)	O/W ratio	Speed (rpm)	Time (s)	Dilution ratio
M01	1	10	2.00	0.10	1,000	60	6
M02	2	20	0.50	0.40	3,000	120	2
M03	0	15	1.25	0.25	2,000	90	10
M04	1	10	0.50	0.25	1,000	120	6
M05	0	15	1.25	0.40	3,000	90	2
M06	2	20	2.00	0.10	2,000	60	10
M07	1	15	1.25	0.40	2,000	60	6
M08	0	10	2.00	0.25	1,000	90	10
M09	2	20	0.50	0.10	3,000	120	2
M10	1	15	0.50	0.40	3,000	120	6
M11	0	10	1.25	0.10	1,000	90	10
M12	2	20	2.00	0.25	2,000	60	2
M13	2	10	1.25	0.40	2,000	120	2
M14	1	20	0.50	0.25	1,000	90	6
M15	0	15	2.00	0.10	3,000	60	10
M16	1	20	0.50	0.25	3,000	60	2
M17	0	15	2.00	0.10	1,000	120	10
M18	2	10	1.25	0.40	2,000	90	6
M19	1	10	0.50	0.40	3,000	60	10
M20	0	20	1.25	0.25	2,000	120	6
M21	2	15	2.00	0.10	1,000	90	2
M22	1	10	2.00	0.40	1,000	60	10
M23	0	15	0.50	0.10	3,000	90	6
M24	2	20	1.25	0.25	2,000	120	2

### 2.3. Microparticles characterization

Microparticles were characterized in terms of particle size by light diffraction using a Mastersizer 2000 (Malvern Instruments, UK). Particle size distributions were characterized by two parameters, the mean particle size and the uniformity (Mastersizer 2000 user manual-Malvern Instruments). Mean particle size was calculated using one of the derived diameters D[m,n] stablished by the British standard BS2955:1993. Particularly, D (v, 0.5) or median of the volume distribution used in this work, corresponds to the size in microns at which 50% of the microparticles population is smaller and 50% is larger. Uniformity is a measure of the absolute deviation from the median, which is calculated following Eq. (1), where *d(x,0.5)* is the median size of the distribution, *d_i_* and *x_i_* are respectively the mean diameter of, and result in, size class *i* (Mastersizer 2000 user manual-Malvern Instruments).

(1) $$\mathrm{Uniformity}=\frac{\sum X_{i\;|d\;(x,\;0.5)-\;d_{i}|}}{d(x,\;0.5)\sum X_{i}}\;\;\;$$

Drug loading (DL) and encapsulation efficiency (EE) were determined by dissolving 3 mg of microparticles in 3 mL of dichloromethane. The drug concentration was afterwards evaluated using a validated spectrophotometric method at 287 nm (Agilent Technologies, USA). DL and EE were calculated using the following equations (Eqs. (2) and (3)):

(2) $$EE\;(\%)=[\frac{\mathrm{actual}\;\mathrm{drug}\;\mathrm{content}\;(mg)}{\mathrm{theoretical}\;\mathrm{drug}\;\mathrm{content}\;(mg)}]\;x\;100\;$$

(3) $$DL\;(\%)=[\frac{\mathrm{actual}\;\mathrm{drug}\;\mathrm{content}\;(mg)}{\mathrm{weight}\;of\;\mathrm{microparticles}\;(mg)}]\;x\;100\;$$

Two different formulations (M10 and M14) were selected as example for small and large microparticles to study in deep their morphology by scanning electron microscopy (SEM) using the EVO LS 15 microscope (Zeiss, Germany). Microparticles were previously coated with silver and images were taken at 500X.

### 2.4. Data mining by neuro-fuzzy logic technology

The complete dataset including the 7 variables as inputs and the values of the 4 formulation parameters studied as outputs: mean particle size (D (v, 0.5)), uniformity, drug loading (DL) and encapsulation efficiency (EE) (Table 2) was modeled using a commercial neurofuzzy logic software, FormRules^®^ v4.03 (Intelligensys Ltd., UK). For continuous outputs as the ones analyzed in this study, the software uses the Adaptive Spline Modeling of Data (ASMOD) algorithm to generate models suitable to elucidate the influence of the different ingredients and operation conditions on the outputs. The models are ‘pruned’ to make them as simple as possible and to generate sets of linguistic IF-THEN rules relatively easy to understand.

**Table 2.** Composition and operation variables followed to develop the polymeric microparticles used to validate the model.

Formulation	[Drug] (%)	[PLGA] (%)	[PVA] (%)	O/W ratio	Speed (rpm)	Time (s)	Dilution ratio
F1	1.0	20.0	1.0	0.25	2,400	60	2.2
F2	1.0	15.0	1.0	0.10	3,000	90	2.0

The training parameters used by FormRules^®^ were the following: ridge regression factor of 1.0 e^−6^, number of set densities: 2, set densities: 2 and 3, maximum inputs per submodel: 4, maximum nodes per input: 15, adapt nodes: true. Among the five fitting statistical criteria offered by FormRules^®^, Structural Risk Minimization (SRM) was used since it gave the highest predictability together with the simplest and more intelligible rules (Shao et al., 2006).

Models’ quality was assessed through the estimation of their predictability and accuracy. The determination coefficient of the Training set (R^2^) was used to establish the predictability and calculated as follows (Eq. (4)):

(4) $$R^{2}=\;[1-\frac{{\sum_{i=1}^{n}\;(y_{i}-y_{i}^{’})}^{2}}{{\sum_{i=1\;}^{n}\left(y_{i}-y_{i}^{’’}\right)}^{2}}]x\;100\text{\% }$$

where *y_i_* is the actual value in the data set, *y_i_*′ is the value calculated by the model, and *y_i_*″ is the mean of the dependent variable. The larger the value of the train set R^2^, the higher the model predictability (Shao et al., 2006). Accuracy was evaluated through an ANOVA that compared predicted and experimental values. Computed *f* ratio values higher than critical *f* values for the specified degrees of freedom indicate no statistical significance between predicted and experimental results and hence, good model performance.

### 2.5. Models’ validation

The models generated by FormRules® are expressed as simple and easy-to-understand rules (Tables S1–S4). These rules were used to select the suitable variables to produce PLGA microparticles with desired characteristics to validate the models. Formulations were prepared under different conditions and compositions (Table 2) and characterized in terms of particle size, uniformity, DL, and EE as previously described.

**Table 4.** Results generated by FormRules^®^ including the critical inputs selected to condition each output and the models quality parameters (determination coefficient and calculated *f*, degrees of freedom (d.f), and α from the ANOVA).

Model	Submodel	Inputs selected by FormRules^®^	R^2^	calcultated *f*	d.f (v1, v2)	α
Mean particle size (μm)	**1**	**[Polymer] × Speed**	92.90	20.35	9, 14	<0.01
Uniformity	**1**	**[Drug] × Speed**	85.26	4.45	13, 10	<0.05
	2	[PVA]				
	3	Time **×** O/W ratio				
	4	Dilution ratio				
EE (%)	**1**	**[Polymer] × O/W ratio**	90.08	3.30	11, 4	0.13
	2	[Drug] **×** Time				
DL (%)	**1**	**[Drug] × [Polymer]**	94.86	87.62	4, 19	<0.01

Inputs with the strongest effect on the corresponding parameters have been bolded.

## 3. Results and discussion

### 3.1. Effect of the studied variables on microparticles properties

**Figure 2.**
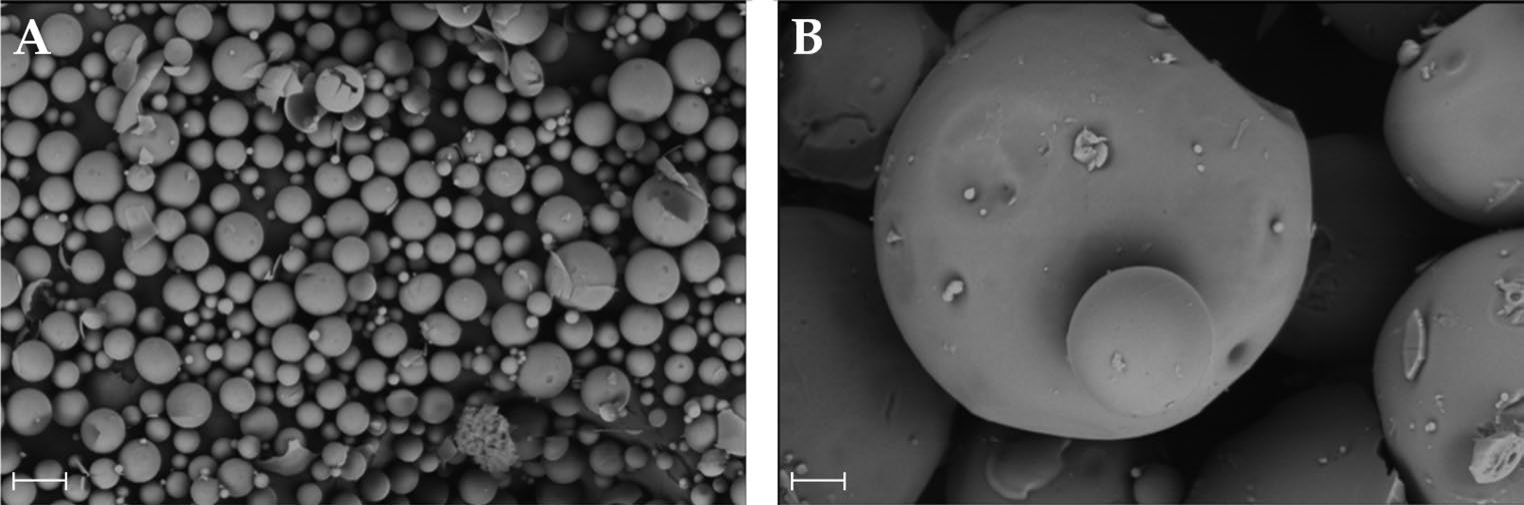
SEM images of Samples M10 (A) and M14 (B) which correspond to the smallest and largest microparticle formulations within the design space. Scale bar 30 µm.

Table 3 shows the results of the experimental design for the 4 parameters studied. Microparticles were always obtained within the established limits of the design space, but they presented variable mean particle sizes, in the of range 17.22 µm–827.60 µm, and disparate distributions (uniformity range 0.21–1.19). Variations in the manufacturing protocol also had an impact on the drug incorporation into the microparticles, as reflected in the EE values (e.g. M10 EE = 47.82%). To evaluate the morphology of the developed microparticles, scanning electron microscopy (SEM) was employed (Figure 2). The SEM images corroborate the differences in size previously observed by light diffraction.

**Table 3.** Experimental values for the studied parameters.

Sample	Mean particle size (µm)	Uniformity	EE (%)	DL (%)
M01	369.72	0.58	59.00	0.56
M02	26.52	0.33	91.05	1.98
M03	21.25	0.26	–	–
M04	193.38	0.63	89.06	1.29
M05	22.33	0.39	–	–
M06	45.53	0.27	104.83	2.08
M07	20.69	0.25	100.41	1.10
M08	431.93	0.57	–	–
M09	33.00	0.25	95.94	2.25
M10	26.22	0.97	46.95	0.47
M11	173.77	0.45	–	–
M12	24.72	0.26	101.22	1.90
M13	33.31	0.50	78.47	1.35
M14	827.60	0.28	95.28	0.85
M15	22.60	0.73	–	–
M16	19.38	0.27	102.35	0.97
M17	212.03	0.38	–	–
M18	17.22	0.21	87.91	1.77
M19	26.47	0.40	101.61	1.05
M20	23.68	0.21	–	–
M21	210.69	1.05	105.70	2.06
M22	185.09	1.19	96.85	1.01
M23	23.61	0.22	–	–
M24	23.89	0.26	98.26	2.02

Despite the small number of experiments, FormRules^®^ managed to model all parameters with high predictability (R^2^ > 85%) and accuracy as shown in Table 4. Additionally, it was able to select the main inputs that explain the variability of each output. Only one variable, dilution ratio, does not seem to exert significant effects on any of the studied parameters. The ASMOD performed by FormRules^®^ splits each model into submodels to point out single or combined effects of variables. The highlighted submodels shown in Table 4 correspond to the inputs with the greatest effect on each output. FormRules^®^ also generates a set of simple linguistic IF-THEN rules for each model together with membership degrees ranging between 0 and 1 (Tables S1–S4). For each of these rules the ‘membership degree’ specifies how a ‘low or high value’ belongs to that fuzzy subset using the technique called fuzzy qualifier (Gago et al., 2011). When precise information is needed, the domains of an output can be observed and quantified, depending on the degree of membership, the specific value predicted by the model (Vysloužil et al., 2014). In this way, the rules generated by FormRules^®^ can be transformed into decision trees (Figures S1–S5).

Microparticle size is a critical quality attribute directly affecting drug release and, therefore, the therapeutic utility of the developed microparticle-based systems (Mensah et al., 2019). The variations in particle size within the database are explained in a 92.9% by a single submodel involving the interaction between the polymer concentration and stirring speed.

**Figure 3.**
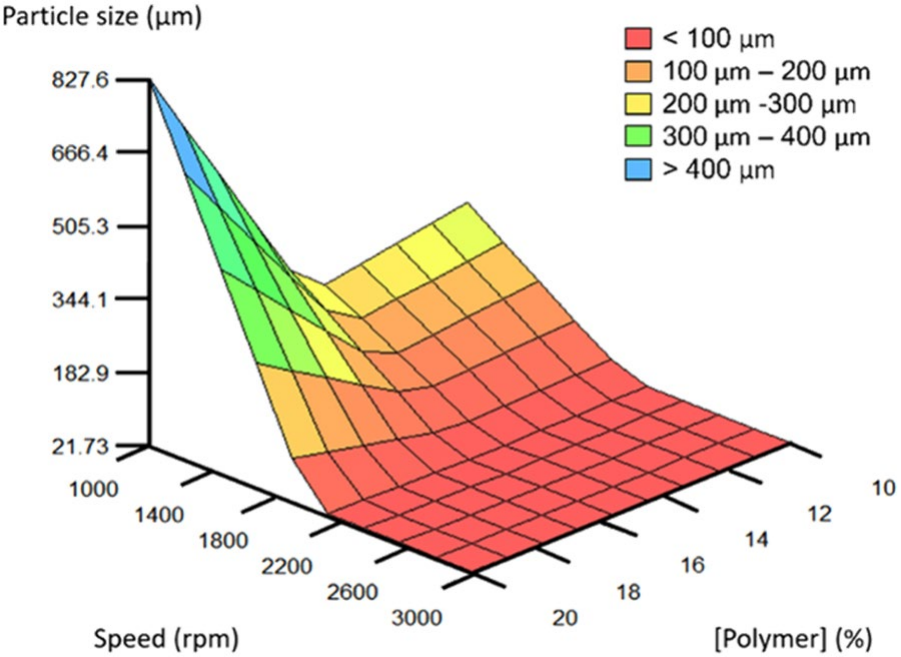
3D plot developed by FormRules^®^ displaying the effect of stirring speed and polymer concentration on the predicted particle size.

The combined effect of stirring speed and polymer concentration in microparticle size is presented in Figure 3. Additionally, the set of IF-THEN rules is shown in Table S1 (Rules 1–9). When the polymer concentration is high (> 17.5%), the use of low stirring speed (< 1,500 rpm) leads to big microparticles (≈ 828 μm) (Figure S1). On the other hand, stirring speeds over 2,000 rpm during the emulsification process allow to obtain always small size microparticles (< 33 μm) regardless of the polymer concentration used (Rules 1–9 Table S1 and Figure S1).

These findings are in agreement with several authors who suggested microparticle size and morphology are conditioned by the energy input during emulsification (Yeo & Park, 2004; Vysloužil et al., 2014). However, no effect of the surfactant concentration (PVA) on microparticle characteristics was observed in our work. In this case, the percentage of polymer in the formulation is critical, which determines the viscosity of the organic phase, and therefore, the energy balance necessary for the formation of the emulsion (Vasileiou et al., 2018). More than 12.5% of PLGA, and a high input of energy (> 1,500 rpm) is necessary to achieve small-sized microparticles (< 25 μm) (Figure S1).

Narrow microparticle size distributions (low uniformity values) are desirable to ensure adequate dosing (Wang et al., 2006; Damiati et al., 2020). Moreover, variations in uniformity can have important consequences on cellular uptake or drug release and compromise the therapeutic effect of the microparticles formulation (Operti et al., 2019). The model obtained for uniformity also shows high predictability (R^2^ > 85%) and accuracy (α < 0.05), but is complex, involving six out of the seven studied variables. FormRules^®^ split the model in four submodels including the interaction between percentage of drug and stirring speed which has the strongest effect, and the interaction of O/W ratio and the stirring time, together with the single effects of surfactant concentration (PVA) and the dilution ratio.

The rules set for this parameter (Rules 1–6 Table S2) shows that submodel 1 has the strongest effect on uniformity and indicates that the size distribution is always narrow unless low stirring speeds together with high drug concentrations or high stirring speeds together with low drug concentrations are used. Moreover, the interaction between the O/W ratio and the stirring time of the emulsification process also influences this parameter, obtaining always narrow particle size distributions unless high O/W ratios (> 0.25) and long emulsification times are used (> 105 s) (Rules 14 Table S2).

**Figure 4.**
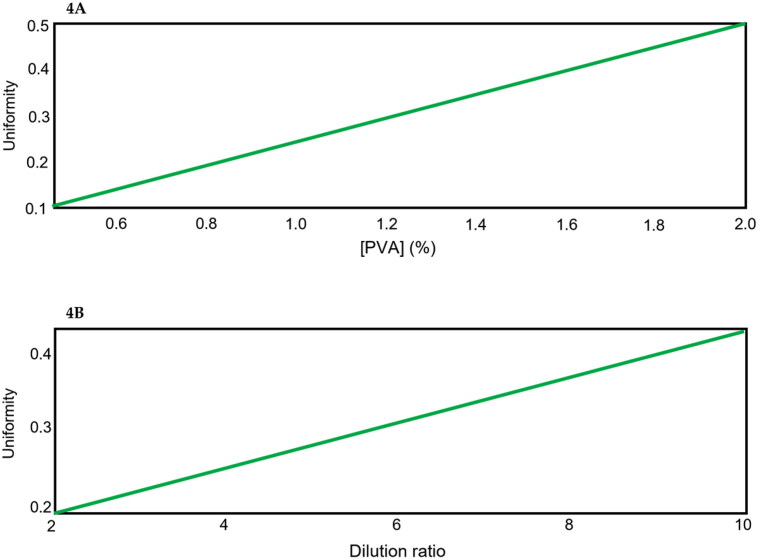
Individual partial effects of the variables on uniformity estimated by the FormRules^®^. (A) effect of surfactant concentration (%), and (B) effect of dilution ratio in size distribution.

Analyzing the surfactant concentration and the dilution ratio individual effects, as shown in Figure 4(A) and Figure 4(B), respectively, it is desirable to use low surfactant concentrations (< 1.25%) and low dilution ratios (< 6) to obtain low size distribution values and consequently, homogeneous size distributions (Rules 7–8 and 15–16 Table S2). On the contrary, Li et al. indicate that when the PVA concentration increases, the particle size decreases, the specific surface area increases and the particles become more uniform, that is, low size distributions are obtained (Li et al., 2022).

**Figure 5.**
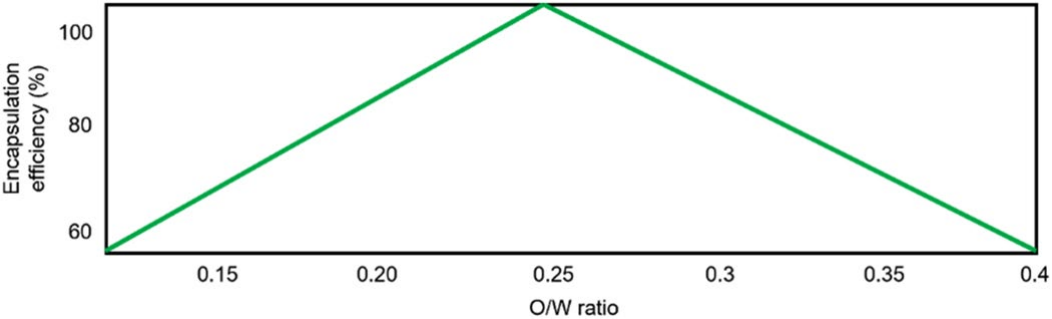
Individual partial effect of O/W ratio on encapsulation efficiency estimated by the FormRules^®^ model.

Two submodels contribute to explain the variations in the encapsulation efficiencies with high predictability (90%). However, probably due to the reduced degrees of freedom, the accuracy could not be adequately demonstrated (Table 4). The variations in EE are mainly explained by the interaction between the polymer concentration and the O/W ratio. In addition, the effect of the interaction between the drug concentration and the stirring time is observed. In agreement with our findings, Yasin et al. have previously reported higher encapsulation efficiencies when greater polymer concentrations were used (Yasin et al., 2021). Our models suggest the use of high polymer concentrations to achieve high encapsulation efficiencies, regardless of the O/W ratios used. This effect has been previously reported by Santoyo S. et al finding that high polymer concentrations increases the emulsion viscosity avoiding drug loss and, therefore, increasing encapsulation efficiency (Rules 4–6 Table S3) (Santoyo et al., 2002). Nevertheless, as derived from Rules 1–3 (Table S3), when low polymer concentrations are used higher encapsulation efficiencies are obtained using medium O/W ratios (≈ 0.2–0.3), namely five times higher the volume of aqueous phase than the one of organic phase (Figure 5). The use of higher O/W ratios has been described to enhance the encapsulation efficiency of PLGA nanoparticles (Hu et al., 2023). Our model shows this effect when O/W ratios between 0.1 and 0.25  were used. However, at higher ratios a decrease in the EE was observed. This non-linear relationship between O/W ratio and EE could be attributed to a detrimental effect of high O/W (> 0.25) over microparticles uniformity resulting in erratic EE (Table S2). Drug concentration also affect this parameter in the range of the database (Rules 7–12 Table S3), obtaining better encapsulation efficiencies when high drug concentrations are used regardless of the emulsification time.

High drug loading is usually desirable to achieve the adequate therapeutic activity. Variations in drug loading within the database are explained in a 94.86% by the interaction between the drug and polymer concentration in the formulation.

**Figure 6.**
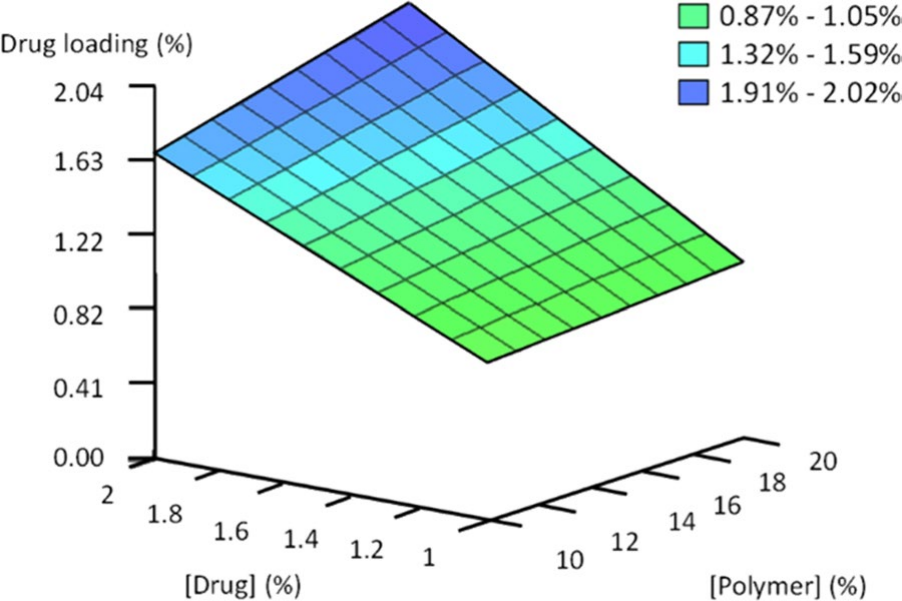
3D plot developed by FormRules^®^ displaying the effect of polymer and drug concentrations on predicted drug loading.

As expected, high drug loading is achieved when high percentage of drug is used. In this situation, the increase in the percentage of polymer improves the drug microencapsulation (Rules 1–4 Table S4) (Figure 6).

### 3.2. Procedure validation

The IF-THEN rules generated by FormRules^®^ allow a better understanding of how the different variables influence the critical attributes of microparticles (Figures S1–S5). To validate the obtained guidelines, the composition and operating conditions were selected to prepare two formulations (F1 and F2) of uniform polymeric microparticles (Table 2). Conditions were selected to obtain small microparticles, with a high encapsulation efficiency (> 70%) and high drug loadings (≈ 1%).

For F1 high polymer concentration (> 17.5%), together with medium (1,500 rpm–2,500 rpm) stirring speeds were used to obtain small particles (Rule 6 Table S1). Moreover, medium O/W ratios (≈ 0.2–0.3) together with low stirring times (< 75 s), and high drug concentration (≈ 1%) were chosen to obtain high encapsulation efficiency (> 70%) (Table 2) (Rule 5 Table S3).

On the other hand, for F2, medium polymer concentrations (12.5%–17.5%), together high (> 2,500 rpm) stirring speeds were selected (Rule 8 Table S1). Furthermore, low O/W ratios (< 0.2) together with medium stirring times (75 s–105 s), and high drug concentration (≈ 1%) were selected to ensure high EE (Table 2).

Other variables that must also be considered are the surfactant concentration and the dilution ratio. Low surfactant concentrations (< 1.25%) and low dilution ratios (< 6) are desirable to obtain a narrow microparticle size distribution. Therefore, 1% of PVA and a dilution ratio of 2.2 and 2 respectively were selected for F1 and F2 according to the obtained models (Rules 7 and 15 Table S2 and Figures S3–S5 at the supplementary data) (Table 2).

Following the conditions established in Table 2, F1 and F2 were produced. Their characteristics are shown in Table 5. The critical microparticles attributes were aligned with the established desirability criteria, since small (≈ 21 µm) and monodisperse microparticles (Uniformity = 0.3) were obtained. These systems showed the desired high encapsulation efficiencies. These results confirm the validity of the obtained neurofuzzy logic models.

**Table 5.** Mean diameter, size distribution, drug loading and encapsulation efficiency of the developed polymeric microparticles following the obtained rules.

Obtained values	Particle size (µm)	Uniformity	DL (%)	EE (%)
F1	21.3 ± 0.4	0.3 ± 0.0	1.0 ± 0.1	100.2 ± 4.9
F2	21.4 ± 0.0	0.3 ± 0.0	1.1 ± 0.0	106.8 ± 3.6

The strategy described in the manuscript is, therefore, suitable to design the procedures to obtain microspheres with the required attributes. In this way, we proved the obtained IF-THEN rules for the selection of the manufacture conditions to obtain microspheres with the required properties.

## 4. Conclusions

The neurofuzzy logic allowed a better decision planning on the formation process of PLGA microparticles by the simple emulsion-evaporation method, even with an extremely reduced experimental design (24 formulations for 7 inputs). Nevertheless, increasing the number of experiments in the experimental design, would increase the degrees of freedom of the model, and therefore, the accuracy. Even so, the strategy derived from the generated neurofuzzy models allow the selection of the composition and the operating conditions to obtain microparticles with specific characteristics and good reproducibility, such as those prepared for the models validation.

This study highlights the importance of the variables involved in the microencapsulation process usually poorly described in literature. Their influence on particle size, size distribution, encapsulation efficiency, and drug loading has been demonstrated. A complete and adequate description of all of them is necessary to develop robust protocols for microparticle manufacturing following the quality by design (QbD) concept, avoiding the lack of reproducibility. In addition, I would advise directing the manuscript to a broader audience and not only to other authors or researchers. The use of the decision trees and model would be of great relevance for industry, pharmacy, and other application fields of PLGA microparticles.

## Supplementary Material

Supplemental Material

## Data Availability

The data that support the findings of this study are available on request from the corresponding author, P. D. R. The data are not publicly available due to intellectual properties protection.
