# Supplementary material for: Rationalized design to explore the full potential of PLGA microspheres as drug delivery systems
Source: Drug Deliv. 2023 Jun 5;30(1):2219864. doi: 10.1080/10717544.2023.2219864 (PMC10243385; doi:10.1080/10717544.2023.2219864)
Supplement: Supplemental Material [file IDRD_A_2219864_SM5896.docx]

**Supplementary Materials**


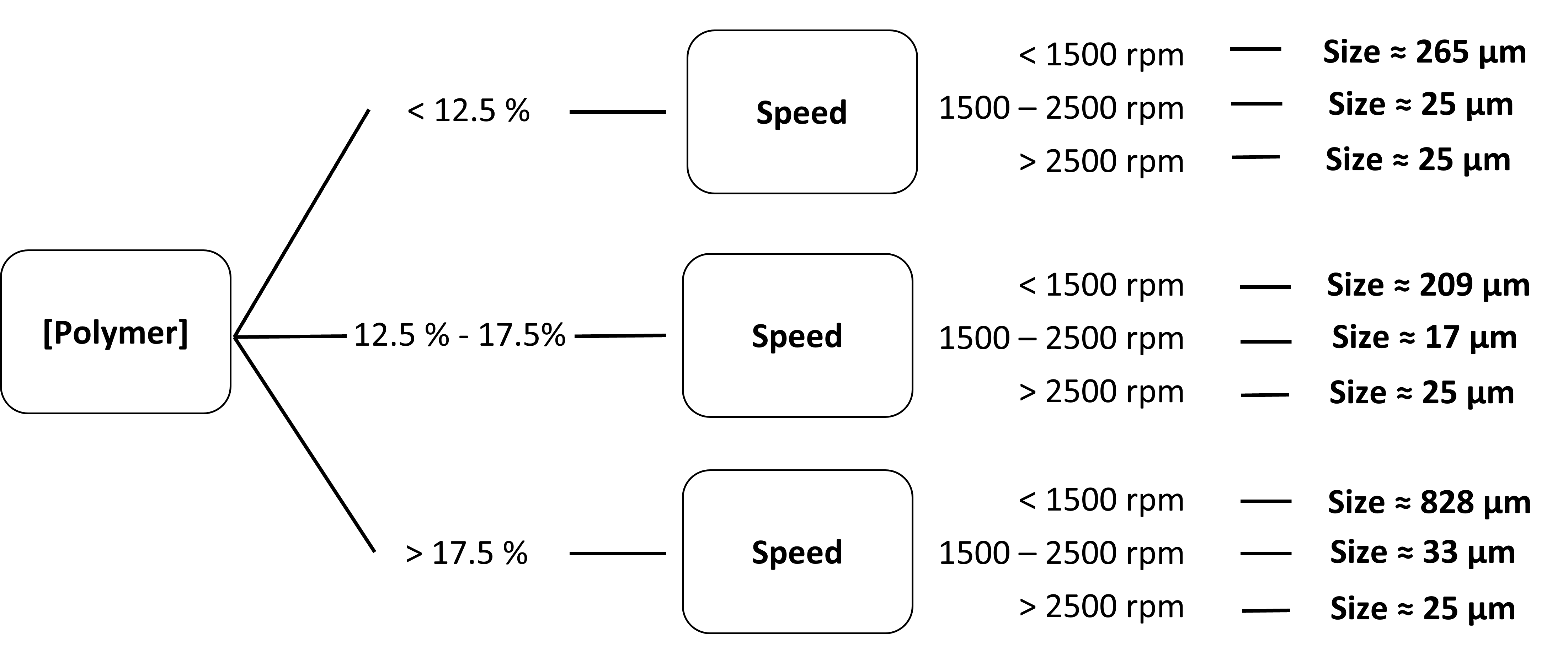


**Figure S1.** Particle size decision tree. (Values of sizes estimated from the rules considering categories and membership degrees).





**Figure S2**. Encapsulation efficiency decision tree. (Values of encapsulation efficiencies estimated from the rules considering categories and membership degrees).





**Figure S3**. Uniformity decision tree when low stirring speeds are used. (Values of uniformity estimated from the rules considering categories and membership degrees).





**Figure S4.** Uniformity decision tree when medium stirring speeds are used. (Values of uniformity estimated from the rules considering categories and membership degrees).





**Figure S5.** Uniformity decision tree when high stirring speeds are used. (Values of uniformity estimated from the rules considering categories and membership degrees).

**Table 1S:** Set of rules from FormRules® for microparticles particle size. Membership degree in parentheses.

| **Rule** | **SubModel:1** |  |  |
| --- | --- | --- | --- |
|  |  |  |  |
| 1 | IF Speed is LOW AND [Polymer] is LOW | THEN Particle size is | LOW (0.69) |
| 2 | IF Speed is LOW AND [Polymer] is MEDIUM | THEN Particle size is | LOW (0.76) |
| 3 | IF Speed is LOW AND [Polymer]is HIGH | THEN Particle size is | HIGH (1.00) |
| 4 | IF Speed is MEDIUM AND [Polymer] is LOW | THEN Particle size is | LOW (0.99) |
| 5 | IF Speed is MEDIUM AND [Polymer] is MEDIUM | THEN Particle size is | LOW (1.00) |
| 6 | IF Speed is MEDIUM AND [Polymer] is HIGH | THEN Particle size is | LOW (0.98) |
| 7 | IF Speed is HIGH AND [Polymer] is LOW | THEN Particle size is | LOW (0.99) |
| 8 | IF Speed is HIGH AND [Polymer] is MEDIUM | THEN Particle size is | LOW (0.99) |
| 9 | IF Speed is HIGH AND [Polymer] is HIGH | THEN Particle size is | LOW (0.99) |

**Table 2S:** Set of rules from FormRules® for microparticles size distribution. Membership degree in parentheses. Rule in blue means the largest positive contribution, while rule in red make the largest negative contribution.

| **Rule** | **SubModel:1** |  |  |
| --- | --- | --- | --- |
| 1 | IF Speed is LOW AND [Drug] is LOW | THEN Size distribution is | LOW (1.00) |
| 2 | IF Speed is LOW AND [Drug] is HIGH | THEN Size distribution is | HIGH (1.00) |
| 3 | IF Speed is MEDIUM AND [Drug] is LOW | THEN Size distribution is | LOW (1.00) |
| 4 | IF Speed is MEDIUM AND [Drug] is HIGH | THEN Size distribution is | LOW (1.00) |
| 5 | IF Speed is HIGH AND [Drug] is LOW | THEN Size distribution is | HIGH (0.68) |
| 6 | IF Speed is HIGH AND [Drug] is HIGH | THEN Size distribution is | LOW (0.62) |
|  | **SubModel:2** |  |  |
| 7 | IF [PVA] is LOW | THEN Size distribution is | LOW (1.00) |
| 8 | IF [PVA] is HIGH | THEN Size distribution is | HIGH (1.00) |
|  | **SubModel:3** |  |  |
| 9 | IF O/W ratio is LOW AND Time is LOW | THEN Size distribution is | LOW (1.00) |
| 10 | IF O/W ratio is LOW AND Time is MEDIUM | THEN Size distribution is | LOW (1.00) |
| 11 | IF O/W ratio is LOW AND Time is HIGH | THEN Size distribution is | LOW (1.00) |
| 12 | IF O/W ratio is HIGH AND Time is LOW | THEN Size distribution is | LOW (0.52) |
| 13 | IF O/W ratio is HIGH AND Time is MEDIUM | THEN Size distribution is | LOW (1.00) |
| 14 | IF O/W ratio is HIGH AND Time is HIGH | THEN Size distribution is | HIGH (1.00) |
|  | **SubModel:4** |  |  |
| 15 | IF Dilution ratio is LOW | THEN Size distribution is | LOW (1.00) |
| 16 | IF Dilution ratio is HIGH | THEN Size distribution is | HIGH (0.88) |

**Table 3S:** Set of rules from FormRules® for microparticles encapsulation efficiency. Membership degree in parentheses.

| **Rule** | **SubModel:1** |  |  |
| --- | --- | --- | --- |
| 1 | IF [Polymer] is LOW AND O/W ratio is LOW | THEN EE is | LOW (1.00) |
| 2 | IF [Polymer] is LOW AND O/W ratio is MEDIUM | THEN EE is | HIGH (1.00) |
| 3 | IF [Polymer] is LOW AND O/W ratio is HIGH | THEN EE is | LOW (0.93) |
| 4 | IF [Polymer] is HIGH AND O/W ratio is LOW | THEN EE is | HIGH (0.92) |
| 5 | IF [Polymer] is HIGH AND O/W ratio is MEDIUM | THEN EE is | HIGH (0.45) |
| 6 | IF [Polymer] is HIGH AND O/W ratio is HIGH | THEN EE is | HIGH (0.66) |
|  | **SubModel:2** |  |  |
| 7 | IF Time is LOW AND [Drug] is LOW | THEN EE is | HIGH (1.00) |
| 8 | IF Time is LOW AND [Drug] is HIGH | THEN EE is | HIGH (0.62) |
| 9 | IF Time is MEDIUM AND [Drug] is LOW | THEN EE is | LOW (0.53) |
| 10 | IF Time is MEDIUM AND [Drug] is HIGH | THEN EE is | HIGH (1.00) |
| 11 | IF Time is HIGH AND [Drug] is LOW | THEN EE is | LOW (1.00) |
| 12 | IF Time is HIGH AND [Drug] is HIGH | THEN EE is | HIGH (0.67) |

**Table 4S:** Set of rules from FormRules® for microparticles drug loading. Membership degree in parentheses.

| **Rule** | **SubModel:1** |  |  |
| --- | --- | --- | --- |
| 1 | IF [Drug] is LOW AND [Polymer]is LOW | THEN DL is | LOW (0.96) |
| 2 | IF [Drug] is LOW AND [Polymer] is HIGH | THEN DL is | LOW (1.00) |
| 3 | IF [Drug] is HIGH AND [Polymer] is LOW | THEN DL is | HIGH (0.74) |
| 4 | IF [Drug] is HIGH AND [Polymer] is HIGH | THEN DL is | HIGH (0.91) |
